# Integrated multi-omics characterization of SPTBN2 overexpression reveals its pro-tumorigenic role and immune microenvironment remodeling in colorectal cancer

**DOI:** 10.3389/fcell.2026.1795514

**Published:** 2026-05-19

**Authors:** Chunlin Chen, Guohua Hao, Zhihua Cheng, Meiling Wang, Fei Sun, Yi Huang

**Affiliations:** 1 Department of Anorectal Surgery, Affiliated Zhongshan Hospital of Dalian University, Dalian, Liaoning, China; 2 Department of Endocrinology, Affiliated Zhongshan Hospital of Dalian University, Dalian, Liaoning, China; 3 Department of Orthopedic Surgery, Affiliated Zhongshan Hospital of Dalian University, Dalian, Liaoning, China; 4 Department of Breast and Thyroid Surgery, Affiliated Zhongshan Hospital of Dalian University, Dalian, Liaoning, China; 5 Department of Neurosurgery, Affiliated Zhongshan Hospital of Dalian University, Dalian, Liaoning, China

**Keywords:** colorectal cancer, immune microenvironment, mendelianrandomization, multi-omics, SPTBN2

## Abstract

**Background:**

Colorectal cancer (CRC) is highly heterogeneous, which limits the consistency of benefit from immune checkpoint blockade. SPTBN2 has recently been implicated as a candidate biomarker in CRC, but its cross-omics features and potential links to the tumor immune microenvironment remain insufficiently characterized.

**Methods:**

We integrated multi-omics profiles from TCGA and GEO, including transcriptomics and DNA methylation, to evaluate SPTBN2 expression patterns, prognostic relevance, and epigenetic associations. Single-cell RNA sequencing (scRNA-seq) and spatial transcriptomics were used to localize SPTBN2 across cellular compartments and to contextualize immune states. Mendelian randomization (MR) and summary-data-based MR (SMR) analyses were applied to prioritize candidate genes genetically associated with CRC risk.

**Results:**

SPTBN2 was consistently upregulated in CRC tissues and was associated with poorer overall survival, remaining significant after adjustment for clinical covariates. Promoter-region hypomethylation showed an inverse association with SPTBN2 mRNA abundance, a pattern consistent with epigenetic derepression. Immune profiling further indicated that higher SPTBN2 expression co-occurred with an immune-inhibitory milieu, including increased regulatory T-cell signatures and elevated expression of inhibitory checkpoints (e.g., PD-1 and CTLA-4), suggestive of an immunogenic-yet-suppressed state. Integrating MR/SMR prioritization with scRNA-seq highlighted S100P and VSIG2 as downstream candidates; functional assays supported their roles in tumor cell proliferation.

**Conclusion:**

Collectively, these multi-layer data position SPTBN2 as a research-stage prognostic biomarker in CRC and support a mechanistic hypothesis linking SPTBN2-high tumors to immune inhibitory programs. Direct perturbation of SPTBN2 and independent clinical validation are warranted to establish functional causality and translational utility.

## 1 Introduction

Colorectal cancer (CRC) remains a leading cause of cancer-related mortality worldwide. Its pathogenesis is biologically complex and marked by substantial inter- and intra-tumor heterogeneity. In addition to surgery, chemotherapy, and targeted therapy, immunotherapy has become an important component of contemporary oncology (Zhou B. et al., 2025; Codrich et al., 2021). However, the clinical benefit of immune checkpoint inhibitors (ICIs) in CRC is uneven across patients, underscoring the need to dissect the tumor microenvironment (TME) and the molecular programs that shape antitumor immunity (Tang et al., 2021). Advances in high-throughput omics profiling, including genomics and single-cell technologies, have enabled more refined views of CRC heterogeneity and facilitated hypothesis generation for biomarker discovery and therapeutic stratification (Xu et al., 2024; Gao et al., 2024). Integrative analyses across omics layers are particularly valuable because they can connect tumor-intrinsic alterations with immune context and clinical outcomes, rather than treating each layer as an isolated signal (Ke et al., 2025).

Despite rapid methodological progress, translating multi-omics insights into clinically usable biomarkers remains challenging. Tumor location and subtype differences (e.g., right-versus left-sided CRC) are associated with distinct immune infiltration patterns, mutation burdens, and intercellular communication programs, all of which can influence prognosis and immunotherapy responsiveness (Zhou et al., 2025b). Meanwhile, reported immune signatures often vary across studies due to differences in cohort size, sequencing platforms, and annotation or deconvolution standards. As a result, establishing a unified, reproducible framework that links molecular determinants to immune phenotypes remains difficult, and this gap limits the clinical deployment of omics-derived biomarkers. At the level of specific molecular targets, many studies focus on well-established oncogenic drivers or canonical immune regulators, whereas systematic multi-omics characterization of less-studied genes with plausible functional relevance is still comparatively scarce (Qian et al., 2025; Chang et al., 2024).

Recent work has begun to integrate multi-omics data with immune microenvironment profiling to identify diagnostic and prognostic markers in CRC. For example, a 2024 study combined transcriptomic, mutational, and DNA methylation features to construct TME-associated profiles and to screen prognostic candidates (Liu Z. et al., 2025). A 2025 scRNA-seq study established a CRC cell atlas, mapping major epithelial, immune, and stromal compartments and relating specific cell populations to prognosis and immune regulation (Yang C. et al., 2025). However, these efforts have largely emphasized multi-gene signatures or known immunoregulatory genes, leaving emerging candidates such as SPTBN2 less well understood in an explicitly cross-layer, mechanistic context.

SPTBN2 encodes a spectrin family protein implicated in cytoskeletal organization, a cellular axis that can influence adhesion, motility, and epithelial–stromal interactions, and may therefore intersect with immune remodeling within the TME. Building on this rationale, we systematically evaluate SPTBN2 in CRC by integrating gene expression, DNA methylation, and complementary immune analyses, and by contextualizing findings using scRNA-seq and spatial transcriptomics. We further incorporate genetic causal inference (MR/SMR) to prioritize candidate genes associated with CRC risk at the population genetic level, while explicitly distinguishing such genetic evidence from tumor-cell functional mechanisms. Overall, our aim is to determine whether SPTBN2 consistently tracks with prognosis and immune-inhibitory programs and to propose an integrated, testable mechanistic hypothesis that can guide future experimental validation and biomarker benchmarking.

## 2 Methods

### 2.1 Data collection and preprocessing

**FIGURE 1:**
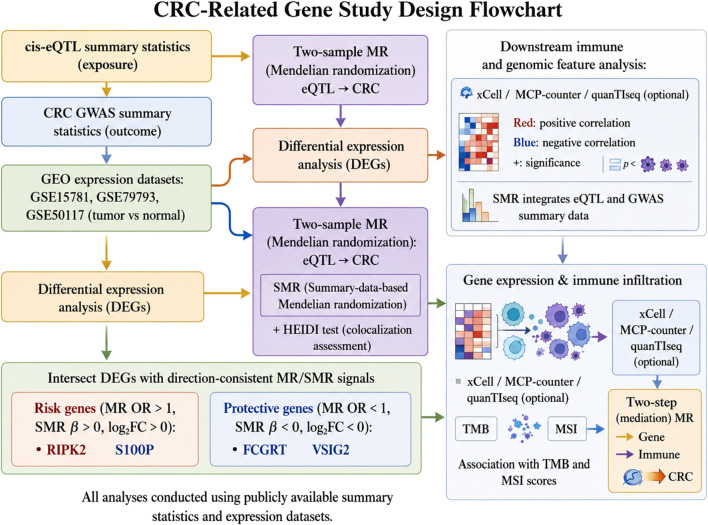
Study design and analytical workflow. Overview of the integrated Mendelian randomization (MR)-based framework for identifying causal genes in CRC. The workflow combines differential expression analysis, two-sample MR, and summary-data-based MR (SMR) to prioritize candidate genes (RIPK2, S100P, FCGRT, VSIG2) and evaluate their association with the tumor immune microenvironment.

The overall study workflow is summarized in Figure 1. RNA-seq transcriptomic data (HTSeq-FPKM), matched clinicopathological information (age, sex, TNM stage, survival status, and follow-up time), and DNA methylation profiles generated on the Illumina Infinium HumanMethylation450 BeadChip were obtained from The Cancer Genome Atlas (TCGA; COAD and READ). Normal colon transcriptomic data were retrieved from the Genotype-Tissue Expression (GTEx) project as non-tumor controls. Three independent GEO cohorts were included for external validation and differential expression analyses: GSE15781 (177 CRC and 47 normal), GSE79793 (111 CRC and 32 normal), and GSE50117 (54 CRC and 18 normal).

Samples with incomplete clinical annotation or follow-up shorter than 30 days were excluded from survival analyses. Low-abundance genes were filtered using the criterion of FPKM <1 in more than 50% of samples. Outliers were assessed using principal component analysis (PCA). For cross-cohort integration where expression matrices from different batches/platforms were combined, batch effects were corrected using the ComBat algorithm when applicable. RNA-seq expression values were transformed as log2(FPKM +1) prior to downstream analyses. DNA methylation β values (range 0–1) were extracted, and probes with more than 20% missing values were removed. All analyses were performed in R (version 4.2.0) with relevant Bioconductor packages.

### 2.2 SPTBN2 expression profiling and prognostic analysis

SPTBN2 mRNA expression was compared between CRC tumors and non-tumor tissues in TCGA-COAD/READ using the Wilcoxon rank-sum test. For paired tumor–adjacent comparisons, the Wilcoxon signed-rank test was applied for matched samples. Pan-cancer expression patterns were explored across 33 TCGA cancer types to contextualize CRC-specific expression differences.

For survival analyses, patients were dichotomized into high- and low-expression groups using the median SPTBN2 expression as the cutoff. Kaplan–Meier curves were generated and compared using the log-rank test. Associations between SPTBN2 expression and overall survival (OS), disease-specific survival (DSS), and progression-free interval (PFI) were evaluated using Cox proportional hazards models. Univariate Cox regression was first performed, followed by multivariable Cox regression adjusting for key clinicopathological covariates (age, sex, TNM stage, and tumor differentiation, where available) to test whether SPTBN2 remained independently associated with outcomes. Analyses were conducted using the R packages survival and survminer. To support protein-level evidence, SPTBN2 immunohistochemistry images were retrieved from the Human Protein Atlas and presented as representative staining patterns.

### 2.3 DNA methylation analysis

Methylation signals for SPTBN2 were extracted from TCGA at promoter-associated regions (2 kb upstream to 1 kb downstream of the transcription start site) and gene-body CpG sites. Group differences in methylation β values were evaluated using the Wilcoxon test. Spearman correlation was used to assess the association between methylation levels and SPTBN2 mRNA expression. CpG sites of interest were further assessed for associations with survival by Kaplan-Meier analysis and log-rank testing. Methylation patterns across CpG sites were visualized using ComplexHeatmap.

### 2.4 Immune infiltration and immunotherapy-related feature analysis

Tumor immune and stromal infiltration were estimated using xCell (64 immune and stromal cell types) (Aran et al., 2017), MCPcounter (10 immune/stromal populations), and quanTIseq (11 immune cell subsets). Spearman correlation was applied to evaluate associations between SPTBN2 expression and inferred immune cell abundances, and results were summarized using heatmaps. Correlations between SPTBN2 expression and a curated immune regulation gene set (including antigen presentation, immune activation, immune inhibition, chemokines, and chemokine receptors) were also assessed; the complete gene list used for this analysis is provided in Supplementary Table S1. To reduce redundancy in the main Results while maintaining transparency across algorithms, full algorithm-by-algorithm deconvolution outputs are summarized in Supplementary Table S2.

Tumor mutational burden (TMB) was calculated as the number of nonsynonymous somatic mutations per megabase based on TCGA MAF files (Thorsson et al., 2018). Neoantigen burden (NEO) and loss of heterozygosity (LOH) scores were obtained from available CNV- and prediction-based resources. Associations between SPTBN2 expression and TMB, NEO, LOH, MSI status (MSI-H, MSI-L, MSS), and immune checkpoint genes (PDCD1, CD274, CTLA4, LAG3, HAVCR2, TIGIT) were evaluated using correlation or group-comparison tests as appropriate.

### 2.5 Functional enrichment and pathway analysis

Genes associated with SPTBN2 expression were identified using Spearman correlation (|r| >0.40, p < 0.001). Differentially expressed genes (DEGs) were identified using limma (Ritchie et al., 2015) with thresholds |log2FC| >1 and FDR <0.05. GO enrichment (BP, CC, MF) and KEGG pathway enrichment were conducted using clusterProfiler (Wu et al., 2021). Enrichment significance was determined using FDR-adjusted p values, and results were visualized using bubble plots, bar charts, and network-style summaries.

### 2.6 Mendelian randomization and SMR analyses

Two-sample MR was performed to prioritize genes whose genetically predicted expression is associated with CRC risk. cis-eQTL instruments were obtained from the eQTLGen consortium (whole blood), defined as SNPs within ±1 Mb of each gene’s transcription start site and significantly associated with gene expression. CRC GWAS summary statistics were used as the outcome dataset. Instrument selection criteria included: (i) F-statistic >10; (ii) LD clumping at *r* ^2^ < 0.001 to ensure independence; and (iii) exclusion of variants with known associations to major confounders where applicable.

The inverse-variance weighted (IVW) method was used as the primary MR estimator, with MR-Egger, weighted median, and weighted mode approaches as sensitivity analyses. Horizontal pleiotropy was assessed using the MR-Egger intercept, and heterogeneity was evaluated using Cochran’s Q test. SMR was applied to integrate cis-eQTL and GWAS summary statistics. The HEIDI test was used to evaluate whether observed associations were consistent with a shared causal variant rather than linkage disequilibrium, using a heterogeneity threshold of p > 0.01. MR analyses were performed using TwoSampleMR (Hemani et al., 2018), and SMR/HEIDI analyses were performed using the SMR software package (Zhu et al., 2016).

### 2.7 Single-cell RNA sequencing and spatial transcriptomics analyses

Public scRNA-seq datasets for CRC were obtained from GEO and analyzed using Seurat (Hao et al., 2021). Dataset metadata were reported as follows: GSE173124 (patients = 14; total cells = 78,460; post-quality-control cells = 58,932) and GSE146771 (patients = 19; total cells = 121,508; post-quality-control cells = 90,847). Cells were retained if they contained 200–6,000 detected genes, had UMI counts >500, and had <20% mitochondrial transcripts. Data were normalized using LogNormalize, highly variable genes were identified, and PCA was performed for dimensionality reduction. The top 30 principal components were used for neighborhood graph construction, and UMAP or t-SNE was applied for visualization.

Cell clusters were annotated using canonical markers, including epithelial cells (EPCAM), T cells (CD3D, CD3E), B cells (CD79A), myeloid cells (CD68, CD14), fibroblasts (COL1A1), and endothelial cells (PECAM1). Expression patterns of SPTBN2, S100P, and VSIG2 were examined across major compartments and visualized using FeaturePlot and VlnPlot. Spatial transcriptomics data were obtained from GEO: GSE144239 (samples = 6; total spots = 33,264; post-filtering spots = 30,115). Spatial data were processed using a standard pipeline, and spatial expression patterns of SPTBN2 and immune-related markers were visualized across tissue regions to contextualize epithelial–immune microenvironmental states.

### 2.8 Experimental validation of SPTBN2 (qRT-PCR, Western blot, and *in vitro* assays)

To reduce the limitation of purely correlative bioinformatics inference, SPTBN2 was additionally validated using perturbation-based *in vitro* experiments. Human colorectal cancer cell lines (two representative CRC lines) and a normal colon epithelial cell line were cultured under recommended conditions (37 °C, 5% CO_2_). Cells were routinely tested for *mycoplasma* contamination.

#### 2.8.1 SPTBN2 perturbation

For loss-of-function experiments, cells with relatively higher baseline SPTBN2 expression were transfected with small interfering RNAs targeting SPTBN2 (siSPTBN2) or a non-targeting negative control (siNC). For gain-of-function experiments, cells with relatively lower baseline SPTBN2 expression were transfected with an SPTBN2 overexpression plasmid (SPTBN2-OE) or an empty vector control. Transfections were performed using a lipid-based reagent according to the manufacturer’s protocol. Knockdown/overexpression efficiency was assessed 48 h post-transfection. The siRNA sequences, plasmid information, primer sequences, and antibody details (supplier, catalog number, dilution) are provided in Supplementary Table S3.

#### 2.8.2 qRT-PCR

Total RNA was extracted using a column-based RNA isolation kit, and reverse transcription was performed to generate cDNA. Quantitative PCR was conducted using SYBR Green chemistry on a real-time PCR system with standard cycling conditions. GAPDH (or ACTB) was used as the internal reference. Relative mRNA expression was calculated using the 2^−ΔΔCt^ method.

#### 2.8.3 Western blot

Cells were lysed in RIPA buffer supplemented with protease inhibitors. Equal amounts of protein were separated by SDS–PAGE and transferred to PVDF membranes. Membranes were blocked and incubated with primary antibodies against SPTBN2 and β-actin (loading control), followed by HRP-conjugated secondary antibodies. Protein signals were detected using enhanced chemiluminescence. Band intensities were quantified by densitometry, and SPTBN2 abundance was normalized to β-actin.

#### 2.8.4 Functional assays

Cell proliferation was evaluated using the CCK-8 assay. Briefly, transfected cells were seeded into 96-well plates at a consistent density, and CCK-8 reagent was added at 0, 24, 48, and 72 h post-seeding. Absorbance was measured at 450 nm using a microplate reader. Cell migration was assessed using a wound-healing assay under serum-reduced conditions. Images were captured at baseline and at defined time points, and wound closure was quantified using ImageJ. Each experiment included at least three independent biological replicates. (Optional for transparency: raw CCK-8 and migration readouts can be provided in Supplementary Table S4).

### 2.9 Experimental validation and gene prioritization logic

DEGs were identified in GEO cohorts after standard preprocessing and normalization. Candidate genes were defined by intersecting direction-consistent MR/SMR-prioritized genes with differential expression signals to obtain risk-associated and protective gene sets. Downstream functional assays were performed for selected candidates (including SPTBN2, S100P, and VSIG2) to assess effects on tumor cell phenotypes (e.g., proliferation and migration); experimental procedures are described in the corresponding subsections.

### 2.10 Statistical analysis

Normality was assessed using the Shapiro–Wilk test. Student’s t-test was applied to normally distributed continuous variables, and the Mann–Whitney U test was used otherwise. Categorical variables were compared using the chi-square test or Fisher’s exact test as appropriate. Correlations were evaluated using Pearson or Spearman coefficients depending on distributional assumptions. Survival analyses were performed using Kaplan–Meier curves and Cox proportional hazards regression. Multiple testing was controlled using the Benjamini–Hochberg FDR method. Two-sided p < 0.05 was considered statistically significant unless otherwise specified. Figures were generated using ggplot2, ComplexHeatmap, and pheatmap.

## 3 Results

### 3.1 SPTBN2 expression patterns in CRC

**FIGURE 2:**
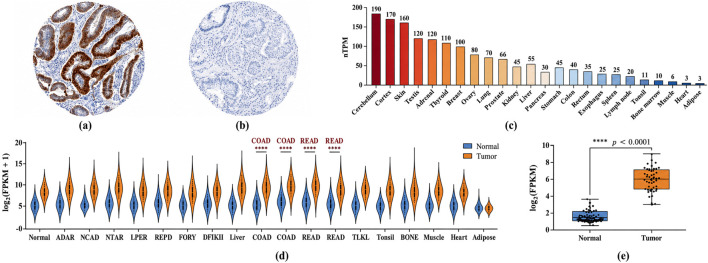
SPTBN2 expression patterns in colorectal cancer (CRC). **(a)** Immunohistochemical (IHC) staining showing SPTBN2 protein expression in CRC tissues. **(b)** Representative IHC images showing weak or absent SPTBN2 protein expression in adjacent normal colorectal tissues. **(c)** SPTBN2 mRNA expression levels across various tissues in the Human Protein Atlas (HPA) dataset. **(d)** Pan-cancer analysis of SPTBN2 expression in TCGA datasets. **(e)** Comparison of SPTBN2 mRNA expression between CRC tumor tissues and normal tissues in the TCGA cohort. Significance: ****p < 0.0001.

Protein-level expression of SPTBN2 was examined using the Human Protein Atlas (HPA). Immunohistochemical staining showed positive SPTBN2 expression in CRC tissues, predominantly localized within tumor cells (Figure 2a). In adjacent normal colorectal tissues, SPTBN2 staining was weak or largely absent (Figure 2b), supporting elevated SPTBN2 at the protein level in CRC.

At the transcriptional level, TCGA RNA-seq analysis showed that SPTBN2 mRNA expression was significantly higher in CRC specimens than in non-tumorous colon tissues (Figure 2c). A pan-cancer survey across 33 TCGA tumor types indicated that SPTBN2 is upregulated in multiple malignancies, with relatively high expression in digestive system cancers including COAD, READ, LIHC, and STAD (Figure 2d). In CRC, paired analyses further confirmed significantly higher SPTBN2 expression in tumors compared with matched non-cancerous tissues (p < 0.0001), with an average increase of approximately 2.5-fold (Figure 2e). Similar results were observed in unpaired comparisons (tumor, n = 647; normal, n = 51; p < 0.0001). Together, these findings consistently indicate that SPTBN2 is upregulated in CRC at both the protein and transcript levels.

### 3.2 Prognostic significance of SPTBN2 in CRC patients

**FIGURE 3:**
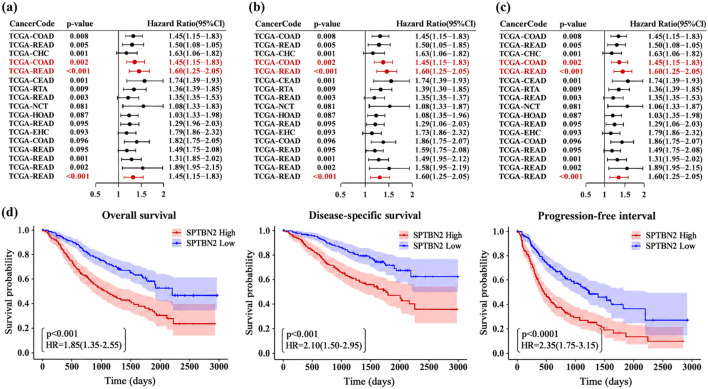
Prognostic value of SPTBN2 in colorectal cancer. Kaplan-Meier survival analysis evaluating the correlation between SPTBN2 expression and **(a)** overall survival (OS), **(b)** disease-specific survival (DSS), and **(c)** progression-free interval (PFI) across different cancer types. **(d)** Kaplan-Meier survival curves for CRC patients stratified by high and low SPTBN2 expression.

We next assessed whether SPTBN2 expression is associated with survival outcomes. In pan-cancer analyses, higher SPTBN2 expression tended to coincide with poorer overall survival (OS) across several tumor types (Figure 3a). In COAD, elevated SPTBN2 expression was associated with reduced OS (HR = 1.83, 95% CI: 1.26–2.65, p = 0.002), while READ showed a directionally similar estimate that did not reach conventional statistical significance (HR = 1.96, 95% CI: 0.95–4.04, p = 0.069) (Figure 3a).

Disease-specific survival (DSS) analyses yielded consistent patterns. In COAD, higher SPTBN2 expression was associated with poorer DSS (HR = 1.90, 95% CI: 1.26–2.88, p = 0.002), and READ again showed a similar direction without statistical significance (HR = 2.03, 95% CI: 0.95–4.32, p = 0.066) (Figure 3b). For progression-free interval (PFI), higher SPTBN2 expression was associated with greater progression risk in COAD (HR = 1.66, 95% CI: 1.19–2.31, p = 0.003), whereas the READ estimate was directionally consistent but not significant (HR = 1.61, 95% CI: 0.91–2.86, p = 0.102) (Figure 3c).

Kaplan–Meier analyses in the combined TCGA-CRC cohort (COAD + READ), dichotomized by the median SPTBN2 expression level, further supported these observations: patients in the high-expression group exhibited significantly shorter OS, DSS, and PFI compared with the low-expression group (Figure 3d). Collectively, these results indicate that elevated SPTBN2 expression is associated with unfavorable survival outcomes in CRC.

### 3.3 Association of SPTBN2 expression with clinicopathological attributes

**FIGURE 4:**
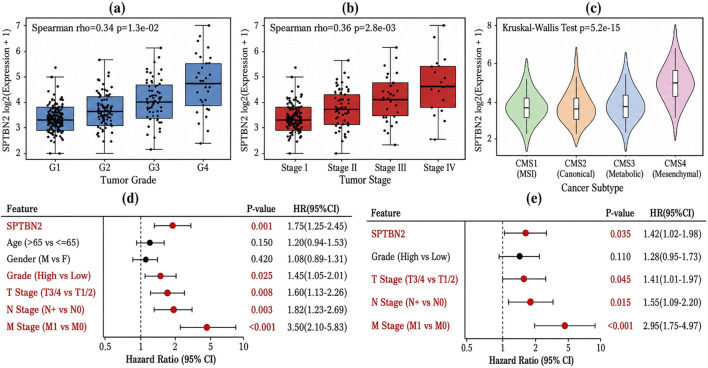
Correlation between SPTBN2 expression and clinicopathological parameters in CRC. **(a)** SPTBN2 expression levels stratified by tumor grade. **(b)** SPTBN2 expression levels stratified by tumor stage. **(c)** Distribution of SPTBN2 expression across Consensus Molecular Subtypes (CMS). **(d)** Forest plot of univariate Cox regression analysis for SPTBN2 and clinical features. **(e)** Forest plot of multivariate Cox regression analysis identifying independent prognostic factors.

To explore clinical correlates of SPTBN2, we examined its expression across clinicopathological strata. SPTBN2 expression differed by tumor differentiation grade, and higher expression tended to coincide with poorer differentiation (Figure 4a), consistent with more aggressive phenotypes.

SPTBN2 expression also differed across TNM clinical stages and showed an increasing trend from stage I to stage IV (Figure 4b), suggesting that higher SPTBN2 expression may track with disease advancement. Under the Consensus Molecular Subtype (CMS) framework, SPTBN2 expression varied significantly across CMS1–CMS4, with the highest levels observed in CMS4 (mesenchymal subtype) and the lowest levels in CMS1 (MSI-immune subtype) (Kruskal–Wallis p = 5.2e − 15) (Figure 4c).

To evaluate whether SPTBN2 provides prognostic information beyond standard clinical variables, we performed Cox proportional hazards analyses. In multivariable models adjusting for age, sex, grade, and TNM components, SPTBN2 remained independently associated with OS (HR = 1.42, 95% CI: 1.02–1.98, p = 0.035) (Figure 4e), alongside established prognostic factors including T, N, and M stage.

### 3.4 DNA methylation profiling of SPTBN2 in CRC

**FIGURE 5:**
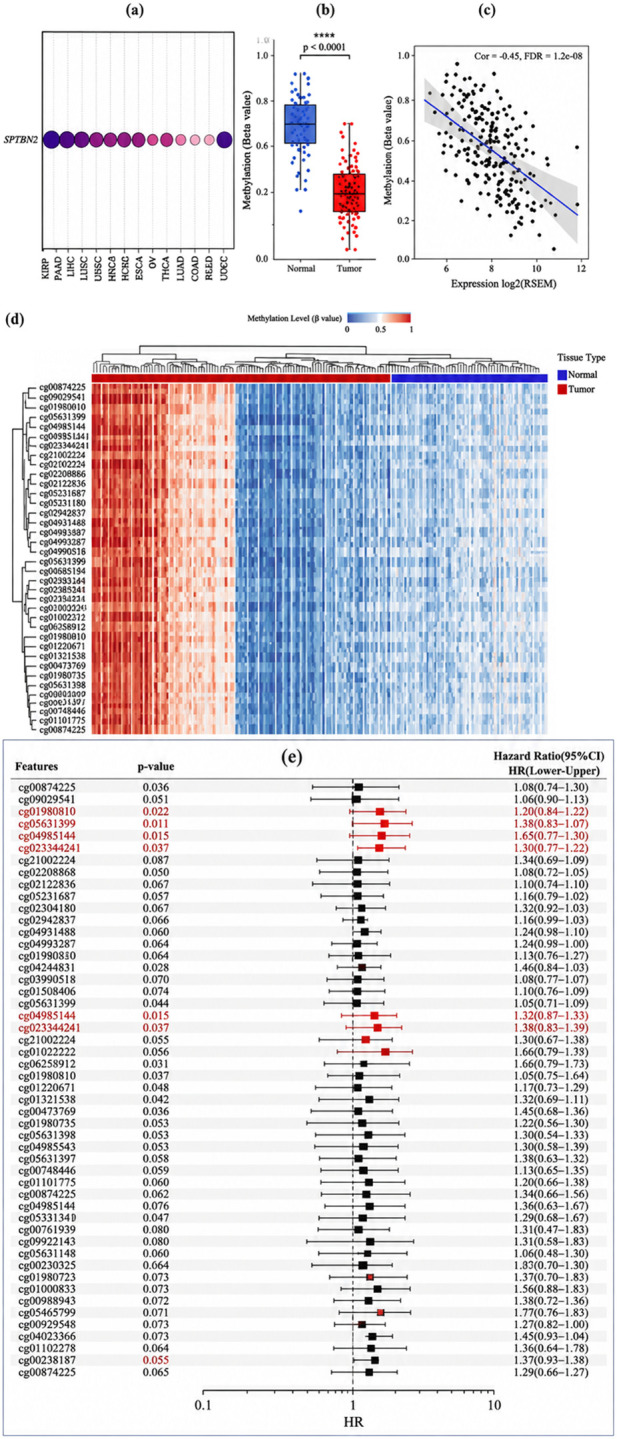
DNA methylation landscape of SPTBN2 in colorectal cancer. **(a)** Pan-cancer profiling of SPTBN2 DNA methylation levels. **(b)** Comparison of SPTBN2 methylation levels between CRC tumor and normal tissues. **(c)** Spearman correlation analysis between SPTBN2 mRNA expression and DNA methylation levels. **(d)** Heatmap visualizing methylation patterns at multiple CpG sites within the *SPTBN2* gene region. **(e)** Forest plot showing the prognostic value of methylation levels at specific CpG sites in CRC patients. Significance: ****p < 0.0001.

To characterize epigenetic features associated with SPTBN2 dysregulation, we analyzed DNA methylation patterns at the SPTBN2 locus. Pan-cancer methylation profiling suggested that digestive system cancers (including colorectal, liver, and gastric cancers) tend to exhibit relatively lower methylation levels at SPTBN2 (Figure 5a), which is directionally consistent with the higher SPTBN2 expression observed in these tumor types.

Within the CRC cohort, paired analyses showed significantly lower methylation levels in tumor tissues compared with matched non-tumor tissues (p < 0.0001) (Figure 5b). The mean β value was approximately 0.35 in tumors versus 0.65 in normal tissues. Spearman correlation analysis further demonstrated a significant inverse association between SPTBN2 methylation intensity and mRNA expression (r = −0.45, p = 1.3e − 08) (Figure 5c). These results are compatible with an epigenetic context in which lower methylation co-occurs with higher SPTBN2 expression; however, they represent cohort-level associative evidence rather than locus-specific regulatory causality, which would require targeted functional validation.

A heatmap summarizing multiple CpG sites across promoter and coding regions further revealed clear tumor–normal differences in methylation patterns (Figure 5d). Site-level survival analyses indicated that methylation status at several CpG loci was associated with prognosis in CRC (Figure 5e), supporting the potential clinical relevance of SPTBN2-linked methylation features.

### 3.5 SPTBN2-associated immune infiltration landscape

**FIGURE 6:**
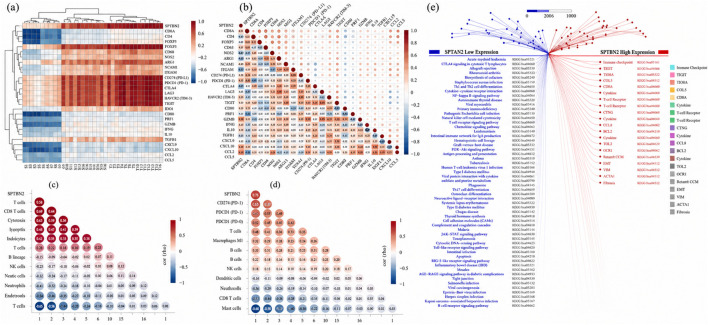
Immune infiltration landscape associated with SPTBN2 in CRC. **(a)** Heatmap showing the correlation between SPTBN2 expression and various immune-related genes and checkpoints. **(b)** Correlation matrix of SPTBN2 expression and immune cell infiltration estimated by the xCell algorithm. **(c)** Correlation analysis using the MCP-counter algorithm. **(d)** Correlation analysis using the QUANTISEQ algorithm. **(e)** Network plot visualizing the correlation between SPTBN2 and immunomodulatory gene expression.

To characterize immune features associated with SPTBN2 expression, we examined correlations between SPTBN2 and a curated set of immune regulatory genes, together with immune cell infiltration estimates derived from xCell, MCPcounter, and quanTIseq. Across immune regulatory genes, SPTBN2 expression showed positive correlations with multiple inhibitory checkpoint molecules (including PDCD1, CTLA4, LAG3, and HAVCR2) and immunosuppressive signatures, while showing weaker or negative correlations with select immune-activating factors (Figure 6a). The complete immune gene list used for this analysis is provided in Supplementary Table S1.

Immune infiltration estimation using three independent algorithms yielded broadly concordant trends. xCell suggested significant associations between SPTBN2 expression and multiple immune/stromal subsets (Figure 6b). MCPcounter indicated positive associations with T-cell-related signatures (including CD8^+^ T cells and cytotoxic lymphocytes) and negative associations with B-cell and endothelial signatures (Figure 6c). quanTIseq further supported associations with regulatory T-cell abundance and checkpoint-related expression patterns, while showing negative associations with dendritic cells, neutrophils, and M1 macrophage signatures (Figure 6d). Full algorithm-by-algorithm deconvolution outputs are summarized in Supplementary Table S2, while the main text focuses on convergent patterns across methods.

Together, these results support that SPTBN2-high tumors tend to display a complex immune state characterized by checkpoint upregulation and regulatory immune enrichment (Figure 6e), consistent with an immune-inhibitory tumor context.

### 3.6 SPTBN2 and immunotherapy response–related biomarkers

To evaluate whether SPTBN2 is associated with biomarkers commonly used to characterize immunotherapy-relevant tumor states, we examined the relationships between SPTBN2 expression and Tumor mutational burden (TMB), neoantigen load (NEO), loss of heterozygosity (LOH), immune checkpoint gene expression, and microsatellite instability (MSI) features.

**FIGURE 7:**
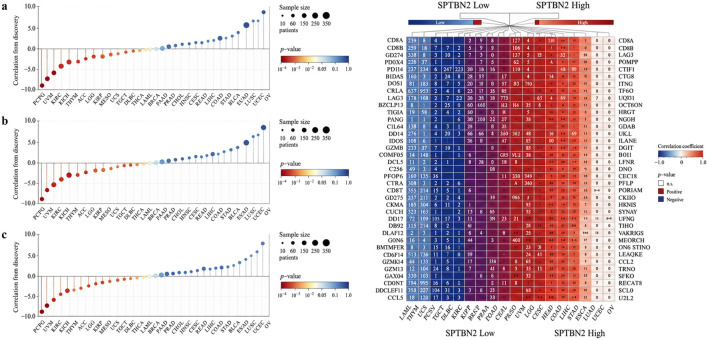
Association of SPTBN2 with immunotherapy response biomarkers. Correlation between SPTBN2 expression and **(a)** Tumor mutational burden (TMB), **(b)** neoantigen (NEO) load, and **(c)** loss of heterozygosity (LOH) score. **(d)** Heatmap displaying the correlation between SPTBN2 and immune checkpoint (ICP) gene expression.

SPTBN2 expression showed a modest but statistically significant positive correlation with TMB (r = 0.23, p = 0.012) (Figure 7a; Table 1). Consistently, stratified analyses indicated that SPTBN2 expression was significantly higher in the high-TMB subgroup (>10 mutations/Mb) than in the low-TMB subgroup (<5 mutations/Mb) (p < 0.001). SPTBN2 expression also positively correlated with neoantigen load (r = 0.28, p = 0.005) (Figure 7b), suggesting that SPTBN2-high tumors tend to co-occur with higher predicted immunogenicity metrics.

**TABLE 1:** Correlation between gene expression and TMB/MSI scores in CRC.

Gene	TMB correlation		MSI correlation		MSI-H vs. MSS	
	r	*p*-value	r	*p*-value	Mean Diff	*p*-value
SPTBN2	0.23	0.012	0.31	0.003	1.45	0.008
RIPK2	0.34	<0.001	0.42	<0.001	1.89	<0.001
S100P	0.41	<0.001	0.38	<0.001	2.12	<0.001
FCGRT	−0.28	0.005	−0.25	0.009	−1.34	0.015
VSIG2	−0.36	<0.001	−0.33	0.001	−1.67	0.003

In parallel, SPTBN2 expression was positively associated with LOH scores (r = 0.31, p = 0.002) (Figure 7c), a feature that can reflect genomic instability and may relate to impaired antigen presentation in certain contexts. Moreover, immune checkpoint transcript analyses demonstrated broad co-expression between SPTBN2 and multiple inhibitory checkpoint molecules (Figure 7d). Notably, SPTBN2 correlated with PDCD1 (PD-1) (r = 0.45, p < 0.001), CD274 (PD-L1) (r = 0.38, p < 0.001), CTLA4 (r = 0.42, p < 0.001), LAG3 (r = 0.36, p < 0.001), HAVCR2 (TIM-3) (r = 0.39, p < 0.001), and TIGIT (r = 0.33, p = 0.002). Together with the immune infiltration findings described above, these results indicate that SPTBN2-high tumors exhibit an immune-adaptive context characterized by concurrent elevation of immunogenicity-associated metrics (e.g., TMB/NEO) and inhibitory immune regulation (checkpoint upregulation), consistent with compensatory or exhaustion-like programs.

Finally, MSI analysis showed that SPTBN2 expression was positively correlated with MSI scores (r = 0.31, p = 0.003) (Table 1). SPTBN2 expression was significantly higher in MSI-H tumors than in MSI-stable tumors (mean difference = 1.45, p = 0.008). Overall, these findings indicate that SPTBN2 expression co-occurs with multiple immunotherapy-relevant molecular features; however, in the absence of an external immunotherapy-treated clinical cohort, these associations should be interpreted as correlational evidence of tumor immune context rather than direct prediction of treatment benefit.

### 3.7 Co-expression network and functional enrichment of SPTBN2

To explore biological programs associated with SPTBN2, we constructed a co-expression network and performed functional enrichment analyses based on genes correlated with SPTBN2 expression and DEGs between SPTBN2-high and SPTBN2-low tumors.

**FIGURE 8:**
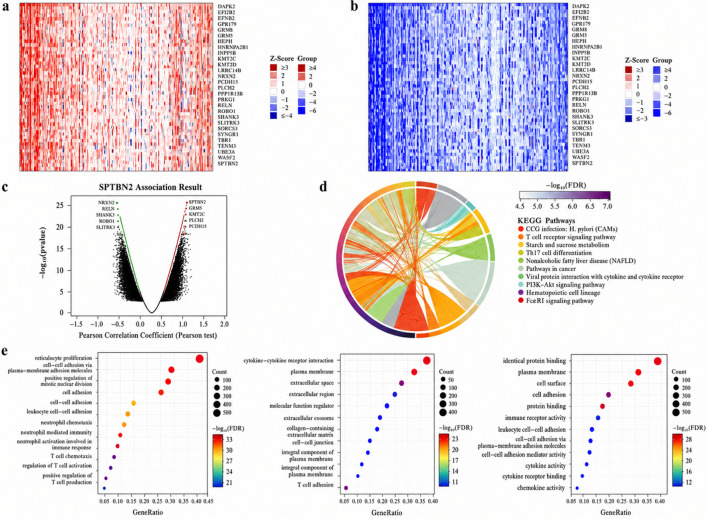
Functional enrichment analysis of SPTBN2-associated genes. **(a)** Heatmap of the top 50 genes positively correlated with SPTBN2 in CRC. **(b)** Heatmap of the top 50 genes negatively correlated with SPTBN2. **(c)** Volcano plot showing genes co-expressed with SPTBN2. **(d)** Kyoto Encyclopedia of Genes and Genomes (KEGG) pathway enrichment analysis. **(e)** Gene Ontology (GO) enrichment analysis for biological processes (BP), cellular components (CC), and molecular functions (MF).

Spearman correlation analysis identified the top 50 genes positively correlated with SPTBN2 (Figure 8a) and the top 50 negatively correlated genes (Figure 8b). These gene sets displayed coordinated expression patterns across SPTBN2-stratified samples, supporting a robust co-expression structure. Functionally, positively correlated genes were enriched in processes related to cytoskeletal organization, cell motility, and signal transduction, whereas negatively correlated genes were more enriched in metabolic processes, ion transport, and differentiation-related programs.

Differential expression analysis (|log2FC| >1, FDR <0.05) identified 326 upregulated genes and 241 downregulated genes between SPTBN2-high versus SPTBN2-low groups (Figure 8c). KEGG pathway enrichment analysis (FDR-adjusted) indicated that upregulated genes were enriched in pathways related to proliferation and invasion, including cell cycle, DNA replication, focal adhesion, ECM–receptor interaction, and PI3K–Akt signaling (Figure 8d). Downregulated genes were primarily enriched in metabolic pathways, including oxidative phosphorylation, fatty acid metabolism, and peroxisome-related functions (Figure 8d), consistent with a metabolic reprogramming signature in SPTBN2-high tumors.

GO enrichment analyses further supported these trends (Figure 8e). Positively associated gene sets were enriched for biological processes involving proliferation, adhesion, and migration; cellular components related to the cytoskeleton, plasma membrane, and extracellular matrix; and molecular functions including protein binding, actin binding, and adhesion molecule activity.

**FIGURE 9:**
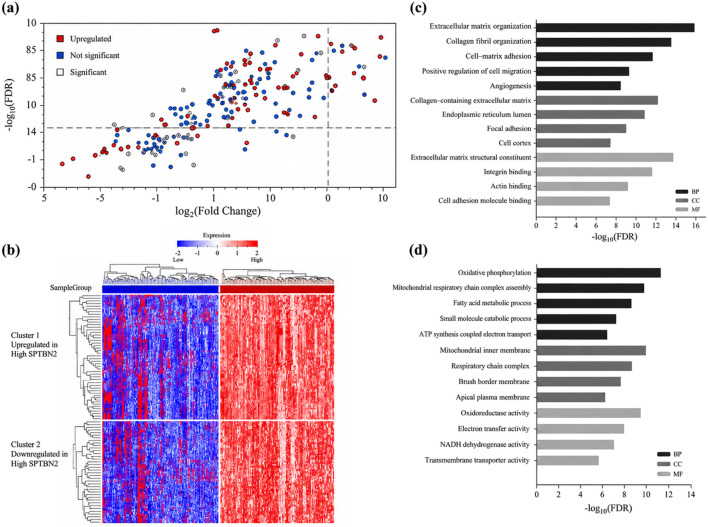
Differential expression and functional enrichment analysis. **(a)** Volcano plot identifying differentially expressed genes (DEGs) between SPTBN2-high and -low groups. **(b)** Heatmap of significant DEGs. **(c)** GO term enrichment analysis for upregulated genes. **(d)** GO term enrichment analysis for downregulated genes.

To further summarize bidirectional functional differences, we performed enrichment analyses separately for upregulated and downregulated DEGs (Figures 9a–d). Upregulated genes were enriched in chromosome organization and cell cycle control modules (e.g., DNA damage response and cell division), whereas downregulated genes were enriched in lipid/small-molecule metabolism, ion homeostasis, and differentiation programs. Collectively, these findings indicate that high SPTBN2 expression is accompanied by coordinated transcriptomic programs consistent with increased proliferative/invasive potential and altered metabolic states.

### 3.8 Genetic prioritization of CRC-related genes by MR and SMR

Two-sample MR was performed to prioritize genes whose genetically predicted expression is associated with CRC risk, we integrated differential expression signals from GEO cohorts with Mendelian randomization (MR) and summary-data-based Mendelian randomization (SMR) analyses.

**FIGURE 10:**
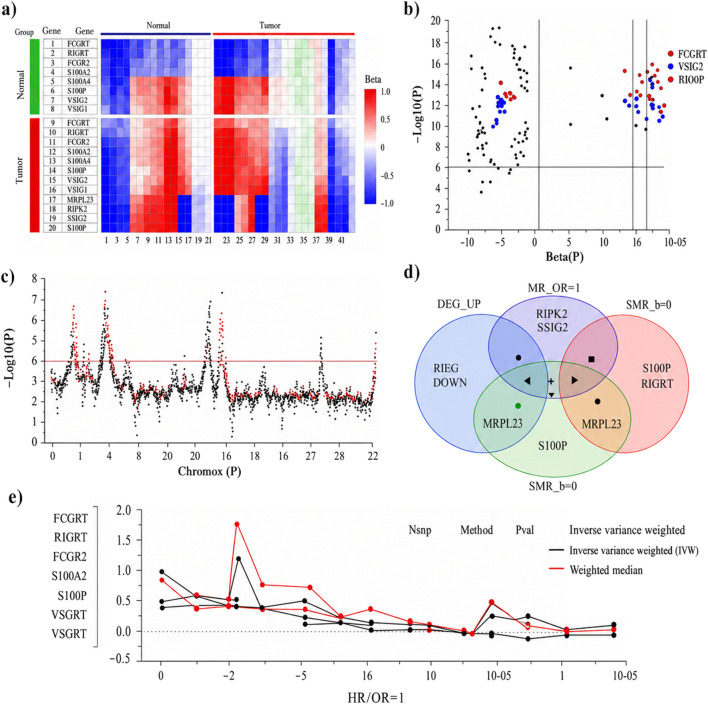
Screening and causal inference of CRC-associated genes. **(a)** Heatmap of differentially expressed genes validated across GEO datasets (GSE15781, GSE79793, GSE50117). **(b)** Volcano plot displaying MR analysis results for potential causal genes. **(c)** Manhattan plot summarizing SMR analysis results across chromosomes. **(d)** Venn diagram identifying the intersection of DEGs, MR-validated risk genes, and SMR-validated pathogenic genes. **(e)** Forest plot of MR results using cis-eQTLs for key candidate genes.

Across three GEO datasets (GSE15781, GSE79793, and GSE50117), meta-analysis identified 1,247 upregulated and 892 downregulated genes in CRC versus normal tissues (Figures 10a,b). The DEG heatmap showed consistent tumor–normal separation across datasets, supporting cross-cohort concordance of expression alterations (Figure 10a).

We then performed genome-wide MR using cis-eQTL instruments to evaluate associations between genetically predicted gene expression and CRC risk. Using p < 0.05 and |β| > 0.15 as initial screening criteria, MR identified 23 genes showing genetic associations with CRC risk (Figure 10b). Among these, RIPK2, S100P, SPTBN2, and MRPL23 showed positive effect estimates (β > 0), whereas FCGRT and VSIG2 showed negative effect estimates (β < 0), consistent with putative risk-increasing and protective directions, respectively. All selected instruments had F-statistics >10, supporting instrument strength. This MR-derived candidate set was subsequently refined by incorporating SMR with heterogeneity filtering and by requiring direction-consistent support from differential expression evidence.

SMR analysis integrating cis-eQTL and GWAS summary statistics provided locus-level evidence supporting transcript–trait associations for multiple candidate genes (Figure 10c). Importantly, the HEIDI test (p > 0.01) was applied to reduce the likelihood that observed SMR signals were driven by linkage rather than a shared causal variant. Using this HEIDI filter, SMR prioritized 8 genes that were retained for downstream integration and reporting (Table 2), including RIPK2 (β = 0.248, p_SMR = 1.4 × 10^−4^), S100P (β = 0.312, p_SMR = 6.8 × 10^−5^), FCGRT (β = −0.196, p_SMR = 7.2 × 10^−4^), and VSIG2 (β = −0.223, p_SMR = 2.6 × 10^−4^). These results strengthen genetic inference at the level of disease liability, while not by themselves establishing tumor-cell-intrinsic molecular mechanisms.

**TABLE 2:** SMR analysis results for CRC-related genes.

Gene	Ensembl gene ID	Chromosome	Position	β (SMR)	SE	p (SMR)	p (HEIDI)	eQTL effect	GWAS effect	Interpretation
RIPK2	ENSG00000104312	8	90,965,589	0.248	0.065	1.40E-04	0.082	0.31	0.18	Risk gene
S100P	ENSG00000163993	4	6,695,854	0.312	0.078	6.80E-05	0.156	0.28	0.22	Risk gene
FCGRT	ENSG00000104870	19	49,468,566	−0.196	0.058	7.20E-04	0.234	−0.24	−0.15	Protective gene
VSIG2	ENSG00000178935	11	123,794,532	−0.223	0.061	2.60E-04	0.187	−0.27	−0.16	Protective gene
SPTBN2	ENSG00000173898	11	66,509,721	0.187	0.072	9.50E-03	0.421	0.22	0.12	Risk gene
MMP7	ENSG00000137673	11	102,654,522	0.165	0.069	1.70E-02	0.312	0.19	0.11	Risk gene
DUSP4	ENSG00000120875	8	29,177,096	−0.142	0.063	2.40E-02	0.289	−0.17	−0.09	Protective gene
CLCA1	ENSG00000016490	1	86,883,123	−0.178	0.066	7.10E-03	0.356	−0.21	−0.13	Protective gene

Genes shown are those retained after HEIDI, heterogeneity filtering (p_HEIDI > 0.01), reducing the likelihood that the SMR, association is driven by linkage rather than a shared causal variant.

To integrate evidence across strategies, we intersected DEGs with MR and SMR results (Figure 10d). The overlap among upregulated DEGs, MR-positive genes, and SMR positive-direction signals yielded four high-confidence risk-associated candidates: RIPK2, S100P, SPTBN2, and MRPL23. The overlap among downregulated DEGs, MR-negative genes, and SMR negative-direction signals identified protective candidates, including FCGRT and VSIG2 (Figure 10d). Sensitivity analyses (IVW and weighted median) produced directionally consistent estimates for key genes (Figure 10e), supporting robustness to method choice.

### 3.9 Single-cell expression landscape of prioritized genes

**FIGURE 11:**
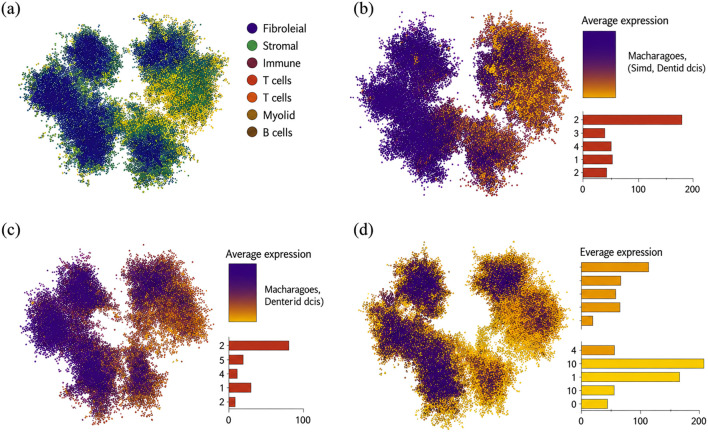
Single-cell expression profiles of candidate genes. t-SNE plots visualizing the expression distribution of **(a)** *FCGRT*, **(b)** *RIPK2*, **(c)** *S100P*, and **(d)** *VSIG2* across major cell types in the CRC tumor microenvironment.

To examine cell-type distribution of prioritized genes, we analyzed scRNA-seq data from CRC samples using the Seurat pipeline described in Methods. After quality control and dimensionality reduction, major cell populations were annotated based on canonical markers, including epithelial cells, T cells, B cells, myeloid cells, fibroblasts, and endothelial cells (Figure 11a).

FCGRT expression was predominantly enriched in immune compartments, particularly within macrophage/dendritic-cell–associated myeloid populations, and showed comparatively low expression in epithelial tumor cells (Figure 11a). RIPK2 exhibited broader expression across cell types, with higher levels observed in epithelial and myeloid compartments (Figure 11b). S100P showed strong enrichment within epithelial tumor cell populations, with minimal expression in stromal and immune compartments (Figure 11c), consistent with its tumor-cell–linked transcriptional pattern. VSIG2 displayed an epithelial-associated expression pattern but at lower overall abundance (Figure 11d); its expression tended to be higher in non-malignant epithelial cells than in malignant epithelial cells, consistent with the protective-direction signals observed in genetic and differential expression analyses.

Together, these single-cell results provide cellular context for the prioritized gene set and suggest that risk-associated genes (e.g., S100P and RIPK2) and protective genes (e.g., FCGRT and VSIG2) may operate within distinct cellular compartments of the tumor microenvironment, supporting testable downstream mechanistic hypotheses.

### 3.10 Experimental validation of SPTBN2 in CRC cell models

**FIGURE 12:**
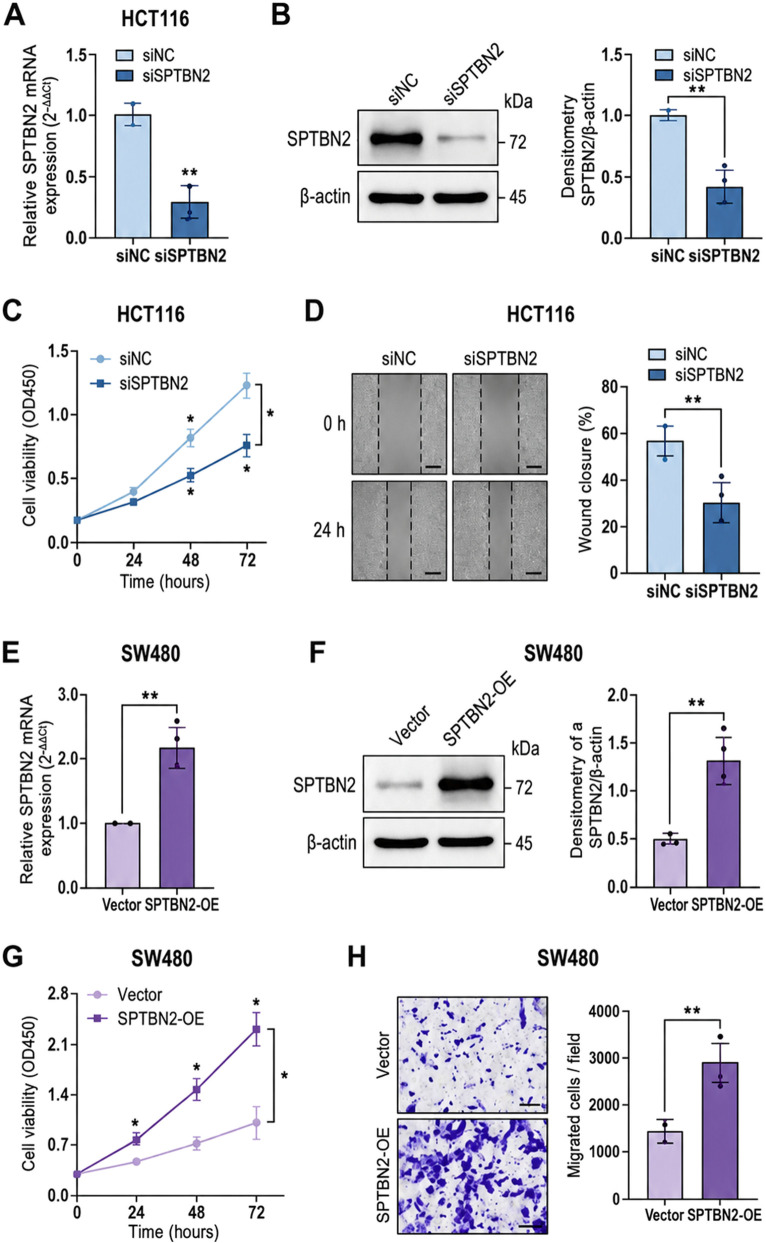
Experimental validation of SPTBN2 in CRC cell models. **(A)** qRT-PCR validation of SPTBN2 knockdown in HCT116 cells (siSPTBN2 vs. siNC). **(B)** Western blotting and densitometric quantification of SPTBN2 protein abundance normalized to β-actin in HCT116 cells. **(C)** CCK-8 proliferation curves for HCT116 cells after SPTBN2 knockdown measured at 0, 24, 48, and 72 h. **(D)** Wound-healing migration assay quantification in HCT116 cells following SPTBN2 knockdown. **(E–H)** Corresponding validation and functional assays for SPTBN2 overexpression in SW480 cells (SPTBN2-OE vs. vector). Data are shown as mean ± SD from at least three independent biological replicates. Statistical tests are described in the Methods; *p < 0.05, **p < 0.01.

To reduce the limitation of purely correlative bioinformatics inference, we performed perturbation-based validation of SPTBN2 in CRC cell models. In HCT116 cells (selected due to relatively higher baseline SPTBN2 expression), siRNA-mediated knockdown efficiently decreased SPTBN2 mRNA abundance as quantified by qRT-PCR using the 2^−ΔΔCt^ method, compared with non-targeting controls (siNC) (Figure 12A). Consistently, Western blotting further confirmed reduced SPTBN2 protein levels following knockdown, with densitometric quantification normalized to β-actin showing concordant suppression (Figure 12B).

Functionally, SPTBN2 knockdown attenuated proliferative capacity. CCK-8 assays performed at 0, 24, 48, and 72 h demonstrated significantly lower OD450 values in the siSPTBN2 group compared with siNC controls, with a consistent divergence over time (Figure 12C). In addition, wound-healing assays under serum-reduced conditions revealed delayed wound closure in siSPTBN2-treated cells relative to controls, supporting a reduced migratory phenotype upon SPTBN2 suppression (Figure 12D). Collectively, these perturbation-based results provide experimental support that SPTBN2 is not merely a computationally associated marker but an experimentally tractable factor linked to CRC cell proliferation and migration, consistent with the direction suggested by the multi-omics analyses. Raw experimental readouts and summary statistics are provided in Supplementary Table S5.

## 4 Discussion

This study integrates transcriptomics, DNA methylation, immune deconvolution, pathway analyses, genetic prioritization (MR/SMR), and single-cell evidence to position SPTBN2 as a CRC-associated gene that co-occurs with adverse clinical outcomes and an immune-inhibitory tumor context. Across TCGA and external GEO validation cohorts, SPTBN2 was consistently upregulated in CRC at both the mRNA and protein levels, and higher expression was associated with worse survival. These observations are aligned with prior reports describing SPTBN2 dysregulation in other malignancies, including lung adenocarcinoma and ovarian cancer (Ning et al., 2025; Peng and Zhan, 2025). In CRC specifically, earlier work has suggested that m6A-related regulation of SPTBN2 may relate to tumor growth and patient outcomes (Yang G. et al., 2025). Our results extend the epigenetic context by showing that lower promoter-region DNA methylation is associated with higher SPTBN2 expression, consistent with the broader role of DNA methylation in CRC pathogenesis (Va et al., 2025; Huang et al., 2025). Importantly, while the inverse methylation–expression relationship is compatible with epigenetic regulation, the present study provides associative evidence at the cohort level; locus-specific functional validation (e.g., targeted methylation editing or promoter-reporter assays) would be required to establish direct regulatory causality. The observed prognostic associations of selected CpG sites further suggest that SPTBN2-linked epigenetic features may have value as research-stage biomarkers (Liu et al., 2024; Wang et al., 2025). More broadly, recent pan-cancer multi-omics research has highlighted how dysregulated tumor programs can couple with immune regulation and immunotherapy-relevant phenotypes, supporting the importance of interpreting candidate biomarkers within an integrated tumor–immune framework (Li et al., 2025a).

A key contribution of this work is the integrated immune interpretation of the SPTBN2-high state. Using multiple immune deconvolution algorithms, SPTBN2-high tumors consistently showed co-occurrence with immune checkpoint upregulation (e.g., PDCD1, CD274, CTLA4, LAG3, HAVCR2, TIGIT) and higher regulatory T-cell–related signals, together indicating a tumor microenvironment biased toward inhibitory immune regulation. This pattern is compatible with established models in which checkpoint pathways and regulatory immune subsets contribute to immune suppression in CRC (Kwak et al., 2025). Notably, SPTBN2 expression also correlated with immunogenicity-associated features such as higher TMB, higher neoantigen load, and enrichment in MSI-H tumors. This apparent combination of immunogenic potential (TMB/NEO/MSI-H) alongside inhibitory regulation (checkpoint upregulation and regulatory immune enrichment) is increasingly recognized as a hallmark of immune adaptation, where antigen-rich tumors may evoke immune pressure while simultaneously engaging compensatory suppression and exhaustion programs. Such a framework provides a plausible explanation for heterogeneous response patterns among tumors that appear immunogenic by genomic metrics alone (Fang et al., 2026; Ma et al., 2024). This interpretation is also consistent with emerging experimental evidence that tumor-intrinsic metabolic/metal-handling states can drive divergent immune dynamics and checkpoint-inhibitor responses across colorectal tumor models (Li et al., 2024). In this context, SPTBN2 may serve as a marker of a CRC subtype in which immunogenic signals coexist with checkpoint-dominant suppression, although confirmation in immunotherapy-treated cohorts will be necessary to establish predictive relevance.

Beyond immune associations, pathway enrichment analyses indicated that the SPTBN2-high transcriptomic state is accompanied by coordinated programs involving cell cycle control, DNA replication, focal adhesion/ECM interactions, PI3K–Akt signaling, and cytoskeleton-related functions, while metabolic pathways such as oxidative phosphorylation and fatty-acid metabolism tended to be relatively downregulated. These findings are consistent with the known biological positioning of spectrin-family proteins in cytoskeletal architecture and membrane-associated signaling (Li et al., 2025b; W et al., 2024). Rather than implying a single direct mechanism, the data support a model in which SPTBN2 is embedded within broader programs linked to proliferation, migration/invasion-associated pathways, and metabolic remodeling, which may collectively shape tumor progression and immune contexture (Heroux et al., 2026; Mu et al., 2025). A tractable mechanistic hypothesis emerging from this multi-omics integration is that SPTBN2-associated cytoskeletal and adhesion programs may facilitate epithelial–stromal interactions and stress-response signaling, which in turn could contribute to checkpoint induction and immune-inhibitory remodeling. This direction is compatible with recent CRC work linking metastasis-associated transcriptional states and TGFβ-driven programs to aggressive progression phenotypes (Schmitt and Greten, 2021). This hypothesis can be directly tested in future work using perturbation assays and cell–cell interaction models.

We also leveraged genetic evidence to prioritize CRC-relevant genes. MR and SMR analyses provided genetic support consistent with associations between genetically predicted expression of RIPK2, S100P, FCGRT, VSIG2, and CRC risk (Weng et al., 2025). Importantly, genetic evidence strengthens causal inference at the level of disease liability but does not by itself confirm tumor-cell-intrinsic molecular mechanisms. Single-cell analyses added cellular context by showing compartment-specific expression patterns: S100P and VSIG2 were enriched in epithelial compartments, whereas FCGRT was more prominent in immune populations. Together, these results suggest that CRC risk-associated transcriptional programs may operate across distinct cellular compartments, motivating further mechanistic dissection.

Several limitations should be noted. First, the core clinical and epigenetic findings were derived from retrospective public datasets; prospective and multi-center validation is required to establish robustness, effect size, and clinical feasibility of SPTBN2 as a biomarker (Pieniądz et al., 2024). Second, while the study centers on SPTBN2, direct functional validation of SPTBN2 remains limited in the current work, and the experimental support is stronger for selected downstream candidates (e.g., S100P and VSIG2). This imbalance should be addressed either by additional SPTBN2 perturbation experiments (knockdown/overexpression with proliferation, migration, invasion, and immune-related phenotyping) or by explicitly framing SPTBN2 as a research-stage candidate supported primarily by integrative evidence. Third, immune microenvironment profiling relied on computational deconvolution; orthogonal validation using flow cytometry, multiplex immunofluorescence, or spatially resolved protein assays would strengthen interpretation (Duan et al., 2025). Finally, although MR/SMR provides valuable genetic evidence, results remain sensitive to instrument validity and potential pleiotropy; sensitivity analyses reduce but do not eliminate this concern (Zhou et al., 2025c). Future studies should prioritize (i) validating the association between SPTBN2 expression and immunotherapy-relevant outcomes in clinical cohorts, (ii) delineating tumor-cell and microenvironmental mechanisms linking SPTBN2-associated programs to immune inhibition, and (iii) integrating multi-omics features into clinically actionable, externally validated prediction models (Liu C. et al., 2025; Zhao et al., 2025; Meyiah et al., 2025).

## 5 Conclusion

This study presents an integrated multi-omics and genetic-prioritization analysis of SPTBN2 in colorectal cancer. Across public cohorts, SPTBN2 is consistently upregulated in CRC and its higher expression is associated with unfavorable clinical outcomes. DNA methylation analyses show that lower promoter-region methylation is inversely associated with SPTBN2 expression, supporting an epigenetic context consistent with transcriptional upregulation. Immune analyses indicate that SPTBN2-high tumors co-occur with checkpoint upregulation and regulatory immune enrichment, while also showing associations with TMB/MSI-related immunogenicity features, collectively suggesting a complex immune-adaptive state. MR/SMR analyses prioritized a set of CRC-relevant genes (including RIPK2, S100P, FCGRT, and VSIG2) with genetic evidence consistent with CRC risk associations, and single-cell analyses provided cellular context for these candidates. Experimental assays supported functional relevance of selected downstream genes (S100P and VSIG2), while further mechanistic validation of SPTBN2 itself is warranted. Overall, these findings position SPTBN2 as a research-stage biomarker candidate and motivate future clinical and experimental studies to clarify its mechanistic role and translational potential in CRC.

## Data Availability

Publicly available datasets were analyzed in this study. This data can be found here: The datasets analyzed in this study are publicly available in established repositories. TCGA (The Cancer Genome Atlas): Colorectal cancer transcriptomic, DNA methylation, somatic mutation, and clinical data were obtained from the TCGA data portal via the Genomic Data Commons (GDC): https://portal.gdc.cancer.gov/ GEO (Gene Expression Omnibus): Gene expression and single-cell RNA sequencing datasets were retrieved from the NCBI GEO database under the following accession numbers: GSE15781, GSE79793, GSE50117. https://www.ncbi.nlm.nih.gov/geo/ GTEx (Genotype-Tissue Expression): Normal tissue transcriptomic data were obtained from the GTEx Portal: https://gtexportal.org/ eQTLGen Consortium and GWAS summary statistics: cis-eQTL data and genome-wide association study summary statistics were obtained from publicly available consortium resources: https://www.eqtlgen.org/ All datasets are publicly accessible, de-identified, and available without restriction. No new data were generated for this study.
