# Supplementary material for: Integrated multi-omics characterization of SPTBN2 overexpression reveals its pro-tumorigenic role and immune microenvironment remodeling in colorectal cancer
Source: Front Cell Dev Biol. 2026 May 19;14:1795514. doi: 10.3389/fcell.2026.1795514 (PMC13226874; doi:10.3389/fcell.2026.1795514)
Supplement: Supplementary file 1 [file Table1.docx]

Supplementary Table S1. Immune regulation gene set used for correlation analyses

| **Category** | **Gene Symbol** |
| --- | --- |
| Antigen presentation | HLA-A |
| Antigen presentation | HLA-B |
| Antigen presentation | HLA-C |
| Antigen presentation | B2M |
| Antigen presentation | TAP1 |
| Antigen presentation | TAP2 |
| Immune activation | CD8A |
| Immune activation | CD8B |
| Immune activation | GZMB |
| Immune activation | PRF1 |
| Immune activation | IFNG |
| Immune activation | CXCL9 |
| Immune inhibition | PDCD1 |
| Immune inhibition | CD274 |
| Immune inhibition | CTLA4 |
| Immune inhibition | LAG3 |
| Immune inhibition | HAVCR2 |
| Immune inhibition | TIGIT |
| Chemokines | CXCL10 |
| Chemokines | CCL5 |
| Chemokines | CCL2 |
| Chemokines | CXCL13 |
| Chemokine receptors | CXCR3 |
| Chemokine receptors | CCR5 |
| Chemokine receptors | CCR2 |
| Chemokine receptors | CXCR5 |

Footnote: Gene symbols follow HGNC nomenclature. Categories are consistent with the manuscript’s predefined immune regulation domains.

Supplementary Table S2. Full immune deconvolution results across xCell, MCPcounter, and quanTIseq

| **Algorithm** | **Cell type / signature** | **Spearman ρ** | **P-value** | **FDR (BH)** |
| --- | --- | --- | --- | --- |
| xCell | Regulatory T cells (Tregs) | 0.31 | 2.1e-06 | 7.8e-05 |
| xCell | CD8+ T cells | -0.18 | 3.6e-03 | 1.4e-02 |
| xCell | Macrophages (M2) | 0.27 | 1.2e-04 | 1.9e-03 |
| xCell | NK cells | -0.15 | 8.5e-03 | 2.6e-02 |
| MCPcounter | T cells | -0.12 | 1.7e-02 | 4.1e-02 |
| MCPcounter | Cytotoxic lymphocytes | -0.20 | 2.9e-03 | 1.5e-02 |
| MCPcounter | Monocytic lineage | 0.22 | 9.8e-04 | 8.7e-03 |
| MCPcounter | Fibroblasts | 0.19 | 4.6e-03 | 2.1e-02 |
| quanTIseq | Tregs | 0.28 | 6.4e-05 | 1.1e-03 |
| quanTIseq | CD8 T cells | -0.16 | 7.9e-03 | 2.7e-02 |
| quanTIseq | M2 macrophages | 0.25 | 3.2e-04 | 3.8e-03 |
| quanTIseq | Neutrophils | 0.14 | 1.1e-02 | 3.1e-02 |

Footnote: Spearman correlation was used to evaluate associations between SPTBN2 expression and inferred cell abundance scores. FDR was computed using the Benjamini–Hochberg procedure within each algorithm’s full set of tested cell types/signatures.
